# Between‐population differences in constitutive and infection‐induced gene expression in threespine stickleback

**DOI:** 10.1111/mec.16197

**Published:** 2021-10-18

**Authors:** Lauren E. Fuess, Jesse N. Weber, Stijn den Haan, Natalie C. Steinel, Kum Chuan Shim, Daniel I. Bolnick

**Affiliations:** ^1^ Department of Ecology and Evolutionary Biology University of Connecticut Storrs Connecticut USA; ^2^ Department of Biology Texas State University San Marcos Texas USA; ^3^ Department of Integrative Biology University of Wisconsin – Madison Madison Wisconsin USA; ^4^ International Institute for Industrial Environmental Economics (IIIEE) Lund University Lund Sweden; ^5^ Department of Biological Sciences University of Massachusetts Lowell Lowell Massachusetts USA; ^6^ Department of Ecology, Evolution, and Behavior University of Texas at Austin Austin Texas USA

**Keywords:** ecoimmunology, evolutionary immunology, fibrosis, host‐parasite interactions

## Abstract

Vertebrate immunity is a complex system consisting of a mix of constitutive and inducible defences. Furthermore, host immunity is subject to selective pressure from a range of parasites and pathogens which can produce variation in these defences across populations. As populations evolve immune responses to parasites, they may adapt via a combination of (1) constitutive differences, (2) shared inducible responses, or (3) divergent inducible responses. Here, we leverage a powerful natural host‐parasite model system (*Gasterosteus aculeatus* and *Schistochephalus solidus*) to tease apart the relative contributions of these three types of adaptations to among‐population divergence in response to parasites. Gene expression analyses revealed limited evidence of significant divergence in constitutive expression of immune defence, and strong signatures of conserved inducible responses to the parasite. Furthermore, our results highlight a handful of immune‐related genes which show divergent inducible responses which may contribute disproportionately to functional differences in infection success or failure. In addition to investigating variation in evolutionary adaptation to parasite selection, we also leverage this unique data set to improve understanding of cellular mechanisms underlying a putative resistance phenotype (fibrosis). Combined, our results provide a case study in evolutionary immunology showing that a very small number of genes may contribute to genotype differences in infection response.

## 1 INTRODUCTION

Parasitic infections undermine host health, and ultimately reduce host fitness (Chomicz et al., 2016; Thompson et al., 2009). Consequently, there are many examples of parasites driving evolution of host resistance or tolerance (Duffy & Forde, 2009; Maze‐Guilmo et al., 2014; Paterson et al., 1998; Van Valen, 1973). In spatially structured host metapopulations (subdivided into isolated populations), this process of immune adaptation may lead to disparate phenotypic solutions, and population divergence (Brunner et al., 2013; Meihls et al., 2013; Weber, Kalbe, et al., 2017). Two populations may evolve to recognize different parasite antigens (e.g., a geographic mosaic of coevolution; Dodds & Thrall, 2009), use different facets of the immune system to achieve the same form of resistance (Brunner et al., 2013; Zhang et al., 2019), or might adopt entirely different strategies (e.g., resistance vs. tolerance; Sagonas et al., 2016). Thus, host populations often evolve divergent immune traits, whether they are faced with geographically varying parasite communities, or even a single widespread parasite.

Vertebrate immunity is a highly derived system consisting of a complex mix of defences and responses adapted to defend hosts against diverse pathogens and parasites (Boehm, 2012). These include constitutively expressed defences (i.e., physical barriers, antimicrobial peptides, phagocytosis, etc Paludan et al., 2021), as well as inducible responses, which are only initiated upon exposure to a potential pathogen (Frost, 1999; Paludan et al., 2021). Inducible responses can further be split into two somewhat overlapping categories: innate responses, which rapidly target broad pathogen categories (e.g., Toll‐like receptor signalling; Kumar et al., 2011), and adaptive responses, which are slower to start but more fine‐tuned to specific pathogens and involve retained pathogen memory (e.g., T and B cell mediated immunity; Mirzaei, 2020). All of these complex responses are potential targets of selection as a result of host‐parasite interactions.

As populations evolve to adapt to a given parasite, to what extent does this evolution lead to between‐population differences in constitutive defences, versus infection‐induced responses? A simple strategy to study this question is to rear different host populations in a common garden environment, then experimentally infect some individuals and measure the resulting phenotypic variation. In particular, transcriptomic measures of gene relative expression are an increasingly common method to evaluate highly multivariate immune traits (Dheilly et al., 2014). One can then statistically partition the resulting variation in gene expression into relative contributions of genotype (G), environment (E, in particular, infection state), and genotype × environment interaction (G × E), to address the above question.

If the populations differ in heritable constitutive defences, then different genotypes raised in a common garden setting may exhibit fixed differences in expression (a genotype effect, G), insensitive to infection. This can happen, for instance, if one population consistently has higher numbers of certain types of immune cells, leading to higher tissue‐level expression of genes unique to that cell type. Numerous studies have documented variation in constitutive immunity as a driver of variation in response to parasites or pathogens (Ali et al., 2012; Evison et al., 2016; Kamiya et al., 2016; Schmitt et al., 2013). For example, genotypes of potato with higher resistance to the pathogen Phytophthora are characterized by increased constitutive expression of immune defences (Ali et al., 2012).

In contrast to constitutive variation, an environmental effect, in this case parasite infection, may trigger consistent inducible immune responses involving a cascade of changes in cell population abundance, signalling, and activation state (i.e., E). For example, social immune responses to mites in honeybees are conserved across multiple independent populations (Oddie et al., 2018). Often these changes can be observed via gene expression (Ingham et al., 2008).

Induced responses to parasite infections may also be variable across populations (genotype by infection interaction, a particular case of more general G × E interactions). In such cases infection may change gene expression more strongly in one population than in another, or even in opposing directions. Plastic or induced responses are known to contribute to variation in parasite resistance (Reeson et al., 1998; Wakelin & Donachie, 1983). In *Drosophila melanogaster*, strains of flies resistant to a pathogenic bacteria are able to induce stronger and quicker innate immune responses than their susceptible counterparts (Okado et al., 2009).

Here, we present an estimate of the relative contributions of genotype, infection, and genotype × infection interactions to between‐population differences in an immune organ's transcriptome, in threespine stickleback, *Gasterosteus aculeatus*. Sticklebacks’ evolutionary history has yielded extensive natural variation in host‐parasite interactions (Bolnick et al., 2020a, 2020b; Poulin et al., 2011; Young & Maccoll, 2017). Ancestral marine stickleback repeatedly colonized freshwater environments during the Pleistocene deglaciation. These new freshwater populations are exposed to a novel helminth cestode parasite, *Schistocephalus solidus*, which is absent in marine environments (McKinnon & Rundle, 2002; Simmonds & Barber, 2016) and rare in anadromous stickleback. Consequently, numerous genetically isolated freshwater populations of stickleback have been independently evolving in response to this freshwater‐associated parasite for thousands of generations (Weber, Kalbe, et al., 2017). The resulting replicated adaptation to *S*. *solidus* resulted in replicated evolution of greater resistance compared to marine fish (Weber, Kalbe, et al., 2017). But adaptation has not been entirely parallel: some populations evolved to drastically suppress cestode growth whereas others evolved to be relatively tolerant, reproducing despite the presence of large cestodes (Weber et al., 2017). Preliminary findings indicate that the growth suppression in these lakes is caused, in part, by the formation of peritoneal fibrotic scar tissue that traps the parasite (Lohman et al., 2017; Weber et al., 2021), and can sometimes kill the parasite. This among‐lake variation in parasite resistance and fibrosis presents a powerful system for investigating relative contributions of G, E, and G × E, adaptations in driving between‐population differences in immune phenotypes (e.g., resistance vs. tolerance).

Specifically, we bred F2 and backcross hybrids between a resistant population (Roberts Lake) and a tolerant population (Gosling Lake) in the laboratory, and experimentally exposed them to *S*. *solidus*. We then assayed infection outcome and stickleback gene expression in an immune organ (pronephros, a.k.a. head kidney). The head kidney is an important site of hematopoiesis and immune cell development in stickleback (Kum & Sekki, 2011). Transcriptomic analyses reveal a mix of G, E, and G × E effects, but expression variation was dominated by constitutive genetic variation (G) with very few G × E interactions. Furthermore, we highlight the transcriptomic signatures and putative immunological mechanisms which underly the putative resistance phenotype, fibrosis peritoneal fibrosis (De Lisle & Bolnick, 2020; Hund et al., 2020; Lohman et al., 2017; Weber et al., 2021). Combining these avenues of research, we provide new evidence suggesting diverse roles of dynamic adaptative responses of hosts to parasites, contributing to improved understanding of host‐parasite evolutionary dynamics.

## 2 MATERIALS AND METHODS

### 2.1 Experimental design

We used minnow traps to capture reproductively mature stickleback from Roberts Lake and Gosling Lake, on Vancouver Island in British Columbia. These populations represent two ends of the natural spectrum of parasite prevalence: high parasite load in Gosling, low in Roberts (Weber, Steinel, et al., 2017). Furthermore, data suggest that Roberts Lake fish have evolved a fibrosis‐ based immune response to suppress parasite growth, which is not present in Gosling Lake fish (Hund et al., 2020; Lohman et al., 2017; Weber et al., 2021). Wild‐caught gravid females were stripped of eggs, which we fertilized using sperm obtained from macerated testes of males from the same lake (within‐population crosses, denoted ROB or GOS) or the other lake (F1 hybrids, RG or GR depending on cross direction). Fish were collected with permission from the Ministry of Forests, Lands, and Natural Resource Operations of British Columbia (Scientific Fish Collection permit NA12‐77018 and NA12‐84188). The resulting eggs were shipped back to Austin, Texas, hatched, and reared to maturity. A subset of these first‐generation laboratory‐raised adults were experimentally infected with *Schistocephalus solidus* cestodes, or sham‐exposed as a control. The resulting infection rates, cestode growth rates, and host immune traits are reported in Weber, Kalbe, et al., 2017, and host immune gene expression is described in Lohman et al. (2017). The remaining laboratory‐reared F1 adults were artificially crossed among families (outbred) to generate F2 hybrids, including both intercrosses (F1 × F1 hybrids), and reciprocal backcrosses (ROB × F1 or GOS × F1). In contrast to Lohman et al. (2017), the fish examined here are second generation laboratory‐raised individuals, allowing us to more confidently exclude maternal effects from wild‐caught parents.

We experimentally exposed 711 one‐year‐old F2 hybrids to *S*. *solidus* cestodes, following standard procedures (Weber, Kalbe, et al., 2017; Weber, Steinel, et al., 2017). Briefly, we obtained mature cestodes from wild‐caught stickleback from Gosling Lake or Echo Lake (Roberts Lake fish do not carry mature cestodes). We obtained the cestodes by dissecting freshly euthanized fish, then paired the cestodes by mass to mate them in nylon biopsy bags in artificial media, mimicking bird intestines where the cestodes typically mate (Wedekind et al., 1998). We collected the resulting eggs, and stored these at 4°C for up to one year. We hatched the eggs and fed them to *Macrocyclops albidus* copepods. The copepods were screened for successful infections after 14 days; then 5 infected copepods were fed to individually‐isolated stickleback, as described in (Weber, Kalbe, et al., 2017; Weber, Steinel, et al., 2017). We filtered the water after the exposure trial to ensure the copepods had been consumed. All F2 hybrid stickleback used in this trial were exposed to *S*. *solidus* (no sham exposures), to maximize infection rate for QTL mapping that has been described elsewhere (Ling et al., 2020). However, only a subset of fish were successfully infected, providing a contrast between infected versus uninfected fish. Prior transcriptomic and flow cytometry data suggest that at the chosen time point (42 days post‐exposure), fish with failed infections are phenotypically similar to sham exposed fish (Lohman et al., 2017). The experimentally infected fish were maintained for 42 days post‐exposure, then euthanized with MS‐222 and dissected to obtain (1) one head kidney (pronephros) for flow cytometry; (2) one head kidney for gene expression analysis, preserved in RNAlater at –80°C; (3) fish mass and length and sex; (4) the mass and number of successfully established cestodes, and (5) the presence or absence of fibrosis. All fish handling was approved by the University of Texas IACUC (AUP‐2010‐00024).

### 2.2 Flow cytometry

Flow cytometry data on head kidney cell population ratios (granulocytes versus lymphocytes) and activity (baseline ROS and oxidative burst) were generated following methods described by Weber, Kalbe, et al. (2017)), Weber, Steinel, et al. (2017)). Data were analysed using flowjo software (Treestar). Populations of granulocytes and lymphocytes were separated by linear forward scatter (FSC) and side scatter (SSC), providing counts of the relative abundance of each cell type. ROS production by granulocytes was measured following protocols for PMA stimulation described in Weber, Kalbe, et al. (2017) and Weber, Steinel, et al. (2017).

### 2.3 RNA extraction and transcriptome sequencing

We extracted RNA from one head kidney using the Ambion MagMAX‐96 Total RNA Isolation Kit, following a modified version of the manufacturer's protocol (see Supporting Information). Each head kidney (hereafter “sample”) was separately placed in lysis/binding solution and homogenized using a motorized pestle. After initial purification using magnetic beads provided by the kit, DNA was removed by adding TURBO DNAse and a second purification with Serapure magnetic beads, leaving only RNA. The RNA yield of each sample was quantified using a Tecan NanoQuant Plate.

RNAseq libraries were constructed using TagSeq methodologies detailed in Lohman et al. (2016) with modifications. After fragmentation of the RNA in a magnesium buffer (NEB Next RNA fragmentation buffer), the RNA fragments were purified using Agencourt RNAClean XP beads. A poly‐dT primer (3ILL‐30TV) was annealed to the poly‐A tail of mRNAs, after which the first cDNA strand was synthesized, which was amplified in a second PCR reaction. The PCR products were purified with Serapure magnetic beads, quantified (Quant‐IT PicoGreen) and normalized (1 ng/µl), after which all libraries were PCR‐barcoded using Illumina i5 and i7 indexes. Fragment size selection occurred via automated gel extraction and final quantification was performed using qubit 2.0. The libraries were sequenced using a hiseq 2500 at the Genomics Sequencing and Analysis Facility of the University of Texas at Austin.

### 2.4 Bioinformatic analyses

We processed TagSeq reads (PCR duplicates removed, adaptors trimmed, low quality reads removed) using the iRNAseq pipeline (Dixon et al., 2015). Reads were aligned to version 95 of the stickleback transcriptome from Ensembl with Bowtie 2 (Langmead & Salzberg, 2012). Samples with less than 500,000 mapped reads were removed from subsequent analyses, resulting in a final *n *= 390. Finally, we annotated transcripts with a blastx comparison to the uniprotkb database (http://www.uniprot.org/help/uniprotkb) with the parameters: max target seqs = 10; evalue = 1e^−5^. Results were filtered to obtain the match with the highest evalue and bit score for each transcript.

### 2.5 Analysis with DESeq2

Raw data, including read count matrixes and metadata, as well as code for all of the following analyses can be found on GitHub (https://github.com/lfuess/TagSeqMS). To test for differential expression, we used the r package DESeq2. Transcripts were filtered to remove those that were not expressed in more than 195 samples (approximately half of the sample set). The remaining 15,354 sequences were tested for differential expression using the following model:

$$Y_{\mathrm{ij}}∼\;\beta_{\text{Batch}}+\;\beta_{\text{Room}}+\;\beta_{\text{Cross}}+\;\beta_{\text{Infection}}+\;\beta_{\text{Fibrosis}}+\;\beta_{\text{ROS}}+\;\beta_{\text{Sex}}+\;\beta_{\text{Cross*Infection}}+\;\beta_{\text{Cross}*\text{Fibrosis}}+\varepsilon_{\mathrm{ij}}.$$

where *Y_ij_* is the count of transcript *I* in individual *j*, *β*_Room_ is a fixed effect with two levels corresponding to the room in which fish were reared, β_Cross_ is a fixed effect with three levels: F2, GBC, and RBC, *β*_Infection_ is a fixed effect with two levels: infected or uninfected, *β*_Fibrosis_ is a fixed effect with two levels: fibrotic or nonfibrotic, *β*_ROS_ is a continuous factor corresponding to measure reactive oxygen production per sample, and β_Sex_ is a fixed effect with three levels: male, female, or unknown (for the few samples where sex could not be identified with confidence). *β*_Batch_ is a random effect corresponding to the lane of sequence sampling. Fibrosis and infection effects were tested independently from one another (i.e., not all fibrotic fish were labelled infected). All *p*‐values were multiple test corrected using a 10% FDR (Benjamini & Hochberg, 1995).

### 2.6 Expression pathway and upstream regulator analyses

Differentially activated biological pathways and upstream regulators were assessed for model factors of interest using the ingenuity pathway analysis software (IPA; Qiagen Inc., https://www.qiagenbioinformatics.com/products/ingenuity‐pathway‐analysis). For each factor of interest, logfold change, unadjusted *p*‐value, and annotation (spID) per transcript was input into the software. Transcripts were then filtered to retain only those with unadjusted *p*‐values < .05. Transcripts with duplicated IDs were averaged for analysis. Pathway analyses were used to identify pathways significantly affected by each factor, and generate corresponding *p*‐values and activation scores (*z*‐scores). Factors tested using IPA were infection, fibrosis, and cross.

## 3 RESULTS

### 3.1 Experimental infection outcomes

Of the 711 fish exposed to cestodes, 268 were infected with *S*. *solidus* after 42 days. Additionally, peritoneal fibrosis was observed in 180 of these fish, 123 of which also had detectable *S*. *solidus* infections. Fibrosis was limited to F2 and RBC fish; no peritoneal fibrosis was observed in any GBC fish. For the random subset of fish used in gene expression analysis: 158 out of 390 were infected with *S*. *solidus*. Moreover, 97 of the fish used in gene expression analyses had peritoneal fibrosis, 70 of which also had detectable *S*. *solidus* infections. Detailed analyses of the infection rates, multivariate immune phenotypes, and genetic mapping of these traits, are presented at length elsewhere (Weber et al., 2021).

### 3.2 Constitutive variation among populations (G)

**FIGURE 1:**
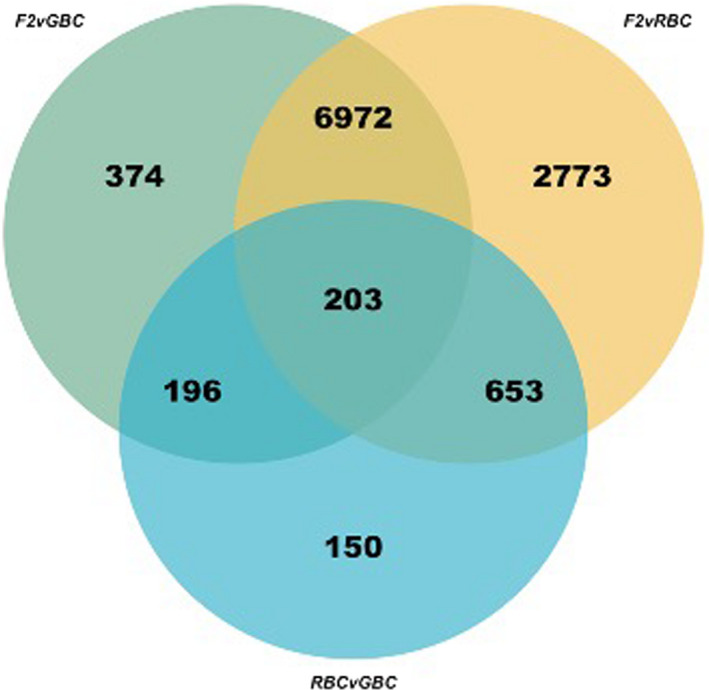
Venn diagram of overlap in significantly differentially expressed genes among all three cross type comparisons

To test for signatures of variation in constitutive immune defences among our populations, we considered baseline variation in gene expression among our three crosses (main effect of host genotype, G, irrespective of infection status). Constitutive gene expression varied considerably. Of the 15,354 genes tested, 11,321 varied constitutively between at least two given cross types: 7745, 10,601, and 1202 transcripts were differentially expressed between F2 versus GBC, F2 versus RBC, and RBC versus GBC, respectively (*p*_adj_ < .10, 10% FDR; Figure 1; Supporting Information S1). A total of 1,216 of these differentially expressed genes have immunological functions (Table 1). However this is not more immune‐related genes than expected from chance (chi‐squared test; *p *= .2627, *χ* ^2^ = 1.2543, *df* = 1).

**TABLE 1:** Example list of genes differentially expressed among cross types with putative functions in immunity. A full list of differentially expressed genes for each contrast can be found in Supporting Information S1

Transcript ID	Annotation	F2 vs. GBC		F2 vs. RBC		RBC vs. GBC	
		LFC	*p*_adj_	LFC	*p*_adj_	LFC	*p*_adj_
ENSGACT00000002677.1	B‐cell receptor CD22	0.718	.00504	1.189	1.81E−8	−0.472	.0702
ENSGACT00000008607.1	Carboxypeptidase N catalytic chain	−0.335	.0551	−0.654	4.93E−6	0.319	.0848
ENSGACT00000021611.1	Complement component C8 alpha chain	0.755	.0863	1.558	1.16E−5	−0.803	.0639
ENSGACT00000012995.1	C‐type lectin domain family 4 member E	1.207	.0379	2.348	5.21E−7	−1.141	.0364
ENSGACT00000024032.1	Gelsolin	1.784	3.05E−9	−0.856	2.16E−4	−0.927	.0974
ENSGACT00000005722.1	Granulins	0.671	3.01E−4	0.331	.0164	0.340	.0562
ENSGACT00000012867.1	H−2 class I histocompatibility antigen, L‐D alpha chain	3.775	3.62E−4	2.026	.0113	1.748	.0798
ENSGACT00000010258.1	Histone acetyltransferase p300	−0.477	.0473	−0.905	4.06E−6	0.428	.0915
ENSGACT00000001096.1	Macrosialin	−0.506	.0168	−0.888	4.44E−7	0.382	.0849
ENSGACT00000002730.1	Transforming growth factor beta activator LRRC33	−0.616	.00347	−1.000	1.21E−8	0.383	.0747
ENSGACT00000024916.1	Ubiquitin‐conjugating enzyme E2 N	0.256	.0927	−0.319	.00785	0.575	5.25E−6

In addition to broad variation among cross‐types in gene expression, numerous pathways and upstream regulators, including many involved in immunity, were also significantly differentially expressed between crosses (*p*_adj_ < .10, 10% FDR), independent of infection status. A total of 240, 313, and 94 pathways, representative of many different immune components, varied in activation state when comparing F2 versus GBC, F2 versus RBC, and RBC versus GBC respectively (Table 2; Figure S1; Supporting Information S2). Additionally, 105, 164, and 22 upstream regulators were predicted to have differential activity between cross comparisons F2 versus GBC, F2 versus RBC, and RBC versus GBC, respectively (Supporting Information S3).

**TABLE 2:** Example list of pathways that were differentially activated among cross types and have putative functions in immunity. A full list of differentially activated pathways for each contrast can be found in Supporting Information S2

Pathway	F2 vs. GBC		F2 vs. RBC		RBC vs. GBC	
	*z*‐score	*p*_adj_	*z*‐score	*p*_adj_	*z*‐score	*p*_adj_
B cell receptor signalling	2.945	.00204	1.889	3.00E−6	0.816	.0398
FLT3 signalling in hematopoietic progenitor cells	2.921	.00398	2.214	3.50E−5	–0.535	.0324
Hepatic fibrosis signalling pathway	5.031	.00138	5.261	4.00E−6	–1.677	.0871
IGF−1 signalling	2.121	.00407	1.852	4.70E−5	0	.0912
IL−2 signalling	1.414	.0589	0.816	.00372	–0.378	.0871
IL−6 signalling	1.761	.0275	1.387	.00105	–0.728	.0776
Leucocyte extravasation signalling	3.960	.0186	3.761	4.00E−6	0.200	.0537
Lymphotoxin β receptor signalling	2.668	.00708	1.225	9.80E−5	–0.378	.0537
NF‐κB activation by viruses	2.600	.0832	2.03	3.10E−5	–0.277	.0479
NF‐κB signalling	3.501	.0427	3.064	3.39E−4	–1.091	.0977
P2Y purigenic receptor signalling pathway	3.317	1.62E−4	2.994	2.24E−7	–1.213	.0324

### 3.3 General response to infection (E)

**FIGURE 2:**
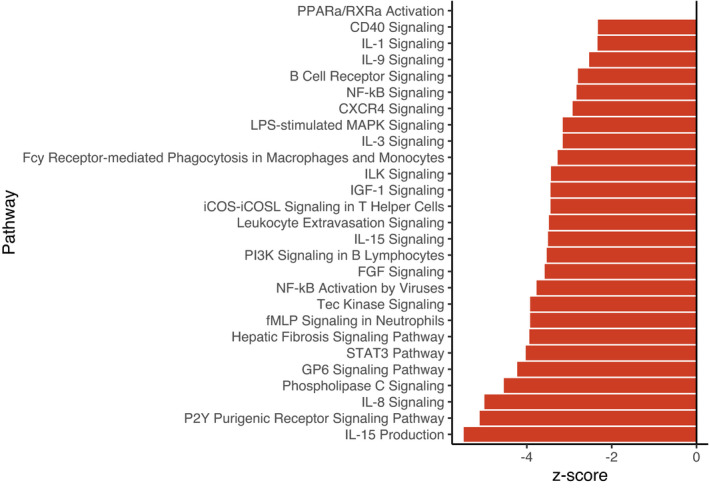
Summary of biological pathways involved in immunity that were significantly activated/inactivated as a result of infection

Host response to infection by a common cestode parasite, *S*. *solidus*, involved multiple genes and pathways. Comparing 158 infected versus 232 uninfected fish (all three cross types), we found 2,369 differentially expressed transcripts (*p*_adj_ < .10, 10% FDR; Supporting Information S1), 2,223 of which were annotated. Of these, 341 transcripts were annotated to encode for proteins involved in immunity. Biological pathway and upstream regulator analyses also indicated broad effects of infection on hosts. A total of 169 pathways were significantly activated as a result of infection (*p*_adj_ < .10, Supporting Information S2). Thirty‐one of these pathways were linked to immunity (Figure 2). All of these significant immune pathways were suppressed (lower relative transcript abundance) in infected fish relative to uninfected fish. Upstream regulator analysis identified 121 regulators which were differentially activated as a result of infection, 27 of which had roles in immunity (Figure S2, Supporting Information S3). Similar to pathway results, regulators displayed broad patterns of immune suppression.

A large fraction of the infection‐responsive genes (1,812 of the 2,369 differentially expressed genes associated with infection, or 76%) also varied in expression between two or more cross types (e.g., genes that exhibit both G and E effects). However, the overlap between the infection‐responsive and constitutively‐different genes was no greater than random expectations (*p *= .2821, *χ* ^2^ = 1.1569, *df* = 1). A particularly noteworthy result is that genes with higher average expression in GBC versus RBC (G effect) are more likely to exhibit a negative response to infection (decreased expression) in both genotypes. Specifically, we found an inverse correlation between directional effect sizes of genotype versus infection main effects for the subset of genes significantly differentially expressed both in infected fish and between RBC versus GBC fish (*p *= .0005439, *df* = 145, cor = –0.2818).

Our results showed little overlap with those from previous transcriptomic study of the response to *S*. *solidus* infection in pure‐cross families (as opposed to hybrids) of these same stickleback populations (Lohman et al., 2017). Fourteen genes were shared between the studies; all but four responded in similar directions (Table 3). Additionally, 76 of the genes that were differentially expressed here were also differentially expressed in a study of liver transcriptome response to *S*. *solidus* infection by stickleback in Germany (Haase et al., 2016). The majority of these genes (~68%; *p *= .001954, *χ* ^2^ = 9.5921, *df* = 1) responded in similar directions (Supporting Information S4). Finally, there was appreciable overlap between the infection‐induced pathways, and the pathways that diverge constitutively between populations. Of the 169 pathways significantly differentially activated as a result of infection, 158 were also differentially activated among two or more cross types (*p *< .001, χ^2^ = 21.528, *df* = 1).

**TABLE 3:** Comparison of infection‐associated significantly differentially expressed genes to results from a previous study (Lohman et al., 2017) using the same two source populations

Transcript ID	Annotation	Current study		Lohman et al.		
		LFC	*p*_adj_	LFC	*p*_adj_	Factor
ENSGACT00000014173.1	Annexin A2‐A	0.811	.00350	1.265	.0730	Infection
ENSGACT00000015567.1	Chromobox protein homologue 8	−0.367	.0210	−0.961	.00699	Infection
ENSGACT00000023042.1	Dopamine beta‐hydroxylase	−0.466	.0698	−2.170	.0662	Infection
ENSGACT00000020041.1	Fibronectin	1.060	3.01E−12	0.887	.0662	Infection
ENSGACT00000004524.1	Glycine‐‐tRNA ligase	0.235	.0205	0.592	.0638	Infection
ENSGACT00000013702.1	Guanine nucleotide‐binding protein‐like 3‐like protein	−0.337	.0637	−0.393	.0929	Infection
ENSGACT00000025278.1	Interleukin−8	0.462	5.64E−4	1.169	.0582	Interaction
ENSGACT00000015612.1	Protein cornichon homologue 1	0.256	.0119	0.455	.0662	Infection
ENSGACT00000008095.1	SID1 transmembrane family member 2	0.262	.0736	−0.557	.0662	Infection
ENSGACT00000018426.1	Sodium channel protein type 2 subunit alpha	−0.299	.0938	−1.104	.0862	Infection
ENSGACT00000011810.1	Sorting nexin−3	0.326	.00612	0.425	.0662	Infection
ENSGACT00000008510.1	Tubulin alpha chain	0.275	.0377	0.978	.0953	Interaction
ENSGACT00000026489.1	Unannotated	−0.441	.0617	−0.823	.0638	Infection
ENSGACT00000008169.1	Unannotated	1.415	2.05E−4	3.239	.0662	Infection

### 3.4 **Variation in induced immune responses between genotypes (G** **× E)**

**FIGURE 3:**
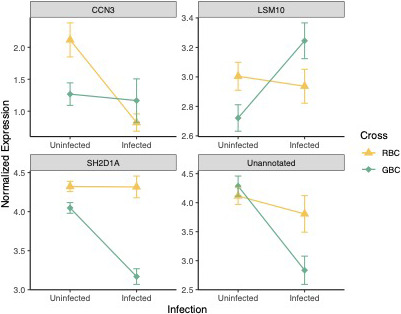
Interaction plot displaying changes in expression of the four genes that responded differentially to infection in RBC vs. GBC fish

A total of 569 genes exhibited interactions between cross type and infection; however, the vast majority of these were only significant when considering differences in response to infection between F2 fish and GBC fish (559/569). Sixty‐four of these genes which responded differentially to infection in F2 versus GBC fish have potential roles in immunity or fibrosis (Supporting Information S1). In contrast, few genes responded differentially to infection when comparing F2 versus RBC and RBC versus GBC fish. Thirteen transcripts responded differentially to infection in F2 versus RBC fish, most of which did not have function in immunity/fibrosis. Finally, examination of differences in response to infection between the two most disparate crosses (RBC/GBC) identified four genes which responded differentially to infection between these crosses, two of which had roles in immunity (Figure 3; Table 4). We were unable to detect significant variation in plasticity in expression of these genes in GBC versus RBC fish alone (one‐tailed Fisher's exact test *p *= .07143).

**TABLE 4:** List of genes that were significantly differentially expressed as a result of the interaction between cross type (RBC vs. GBC) and infection

Transcript ID	Annotation	RBC vs. GBC	
		LFC	*p*_adj_
ENSGACT00000015170.1	CCN family member 3	4.866	1.45E−6
ENSGACT00000018024.1	Unannotated	–2.159	.0986
ENSGACT00000024555.1	SH2 domain‐containing protein 1A	–1.202	.0473
ENSGACT00000003040.1	U7 snRNA‐associated Sm‐like protein LSm10	1.157	.0473

### 3.5 Transcriptomic signatures of fibrosis

**FIGURE 4:**
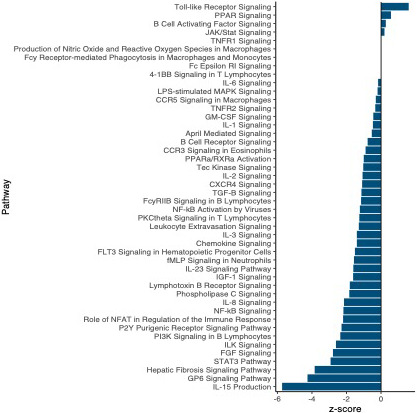
Summary of biological pathways involved in immunity that were significantly activated/inactivated as a result of fibrosis

**FIGURE 5:**
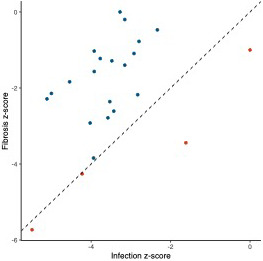
Comparison of activation (*z*‐score) of immune‐related pathways that were significantly activated/inactivated as a result of both fibrosis and infection. Dotted line represents equivalent activation as a result of both factors; points in blue are more activated in fibrotic fish; points in red are more activated in infected fish

We also leveraged these data to identify changes in expression associated with a putative resistance phenotype: fibrosis. We identified strong transcriptomic signatures of fibrosis: 5826 genes were differentially expressed in fibrotic fish compared to those not displaying the fibrosis phenotype, 840 of which have putative roles in immunity and fibrosis. Similar to the effects of infection, the majority of these genes were downregulated in fibrotic fish. Pathway and upstream regulator analyses also revealed significant downregulation of immune‐related processes in fibrotic fish (Figure 4; Figure S2). Many (257) biological pathways were significantly differentially activated in fibrotic fish, 53 of which are related to immunity, defence, or fibrosis responses. Most of these immune‐related pathways were also significantly differentially activated in fish infected with *S*. *solidus*. However, these shared pathways were almost always downregulated to a greater degree in infected fish, compared to fibrotic fish (Figure 5).

## 4 DISCUSSION

### 4.1 Variation in constitutive expression of immune defences across genotypes (G)

Here we document significant variation in gene expression as a result of both genetic variation between two populations, and cestode infection (an environmental effect), and their interaction. By far the dominant source of variation in gene expression arose from heritable differences between genotypes (Roberts Lake backcrosses, F2 hybrids, and Gosling Lake backcrosses), irrespective of infection status. Given the large number of heritable differences in gene expression between the populations, it is challenging to interpret these differences in a functional framework. The expression differences may be adaptive or neutral. If adaptive they may serve any of a vast number of functions given the many phenotypes that differ between these lakes including skeletal morphology, size, behaviour, male breeding colours, diet, microbiota, diverse species of parasites besides *S*. *solidus* (Bolnick et al., 2020b; Ling et al., 2020; Snowberg et al., 2015). Constitutive expression differences do contain many immune genes (Table 2), but these are not significantly overrepresented. Consequently, we cannot confidently claim that these differences in constitutive gene expression have a role in adaptation to *S*. *solidus* or differences in immunity between the fish populations.

Despite the lack of significant enrichment of immune traits among the many genes showing heritable constitutive divergence in expression, there were several notable trends in variation in immune gene expression among our crosses. For example, eight genes related to the complement cascade varied significantly between RBC and GBC fish; these genes were almost exclusively expressed higher in RBC fish. Similarly, 17 and 34 complement genes were differentially expressed between F2 versus GBC and F2 versus RBC respectively, though with mixed patterns of expression. Complement cascade proteins have been shown to contribute to parasite defences in other species of fish (Duan et al., 2021; Xie et al., 2021). When considering pathways and upstream regulators, B cell receptor signalling was activated in GBC compared to RBC fish whereas RBC fish activated both IL‐7 and IL‐3 signalling pathways compared to GBC fish. B cells are an essential component of fish immunity (Sunyer, 2012), including serving roles in the defence against parasite‐induced proliferative kidney disease in salmonids (Abos et al., 2018; Bailey et al., 2017). Additionally, localized expression of interleukins is known to play a role in fish‐parasite interactions (Perez‐Cordon et al., 2014). In vertebrates, IL‐3 specifically plays roles in development of essential antiparasitic immune cells such as mast cells and basophils (Lantz et al., 1998). Finally, RBC fish had higher activation of upstream regulator TGF‐beta 1 compared to GBC fish. TGF‐beta 1 is an important regulator of fibrosis (Gressner et al., 2002), which is an important parasite resistance phenotype in stickleback (see later sections; Weber et al., 2021). Consequently, while not statistically overrepresented as a gene ontology category, some of the observed differences in constitutive expression of immune genes may indeed contribute to divergence in parasite responses.

### 4.2 Conserved responses to a cestode parasite (E)

The next most important factor in our data was a main effect of infection (an environmental effect) irrespective of genotype. These genes represent a multigenic and multipathway response to infection representative of a diversity of immune components. This type of multifaceted response is common across systems: host response to parasite infection often involves multiple arms of the immune system (Anthony et al., 2007; McRae et al., 2015; Medzhitov, 2007; Nakada‐Tsukui & Nozaki, 2016). Genes involved in antiviral responses (zinc finger CCCH‐type antiviral protein 1; Zhang et al., 2020), T cell functioning (C‐type lectin domain family 4 member E; Lu et al., 2018), and Toll‐like receptor signalling (Toll‐like receptor 8; Cervantes et al., 2012) were characteristic of this shared response. Pathway analyses corroborated individual gene results, identifying a diversity of immune pathways which were differentially activated in response to infection regardless of host population. Furthermore, these analyses revealed clear patterns of suppression of these diverse immune pathways at the time point of sampling. Pathways involved in inflammation and chemotaxis such as IL‐8 signalling (Kany et al., 2019), PPARα/RXRα activation pathway (Bougarne et al., 2018), and CXCR4 signalling (Pawig et al., 2015) were commonly suppressed. A number of pathways related to phagocytosis were also suppressed including Fcy receptor‐mediated phagocytosis in macrophages and monocytes, phagosome formation, and phagosome maturation. Upstream regulator pathway analysis confirmed these patterns of broadscale reduction in immunity in infected fish. Antifibrotic regulator hepatic nuclear factor 4‐alpha, HNF4A (Ji et al., 2018; Yue et al., 2010) was significantly activated as a result of infection. Intriguingly, HNF4A is also a major target of natural selection driven differences between Roberts and Gosling Lake, falling within the quantitative trait locus for production of granulomas that encase and frequently kill the cestode (Weber et al., 2021). Several important adaptive immune components such as interleukin‐4 (IL4; (Heeb et al., 2020) and IgE complex (Galli & Tsai, 2012) were also suppressed in infected fish. Together, gene expression and pathway analyses reveal wide patterns of immune suppression associated with infection of *G*. *aculeatus* with *S*. *solidus*. This suppression in infected fish could arise from three processes: initially immune‐suppressed fish might have been more vulnerable to infection, fish observed to be infected might be those that have adapted tolerance rather than resistance strategies, or successfully established parasites may be actively suppressing host immunity. Time‐series analyses of individual host expression could distinguish these alternatives, but is not practical because fish must be euthanized to obtain head kidney samples.

Comparison of the results of our study to previous transcriptomic analyses of stickleback response to *S*. *solidus* revealed limited overlap in infection‐responsive genes. When comparing our results to those from a previous study of fish from pure‐cross families generated from the same populations (Lohman et al., 2017) we found limited shared genes, though the patterns of expression within this small group were mostly congruent (Table 3). Two shared genes of particular interest were interleukin 8 and fibronectin (also a genomic target of selection within one of the resistance QTL mapped by Weber et al. (2021). Infection was associated with higher expression of the extracellular matrix glycoprotein, fibronectin, in both studies. This is potentially indicative of induction of fibrotic resistance phenotypes, as fibronectin production is associated with increased fibrosis (Bochaton‐Piallat et al., 2016; Duffield et al., 2013; Valiente‐Alandi et al., 2018), which can encapsulate the parasite in a web of scar tissue that limits its growth (Weber et al., 2021) possibly by constraining movement and foraging. In contrast, interleukin 8, an important immune chemokine, showed slightly disparate patterns of expression across the two studies. This transcript increased in expression in infected fish in our study, regardless of cross type, but in the previous study, responded differently to infection across populations (G × E interaction). Increased expression of IL8 was associated with infection in fibrosis‐prone pure Roberts fish, while in nonfibrotic Gosling fish IL8 expression was lower in infected individuals (Lohman et al., 2017). The contrast between this previous G × E interaction, and our present E effect, may simply be a matter of imperfect statistical power, or may be an outcome of epistatic interactions that impact pure parental genotypes differently than hybrids. Comparing our results more broadly to studies of stickleback from distant populations (Germany: Haase et al., 2016) revealed a small group of conserved response genes, the majority of which responded in similar directions. However, these two studies used *G*. *aculeatus* and *S*. *solidus* from different continents and different tissue types, potentially explaining the lack of signatures of conservation of infection response. Increasing study of host‐parasite dynamics across the circum‐polar range of stickleback will aid in identifying any shared mechanisms of infection response in this system.

### 4.3 **Variation in response to inducible responses across genotypes (G** **× E)**

A third potential source of variation in response to parasite exposure is divergence in inducible immune responses across genotypes (genotype × infection interactions). Consequently, we searched for genes which responded differentially to infection among our three cross types. Previous analysis of pure F1 ROB versus GOS fish showed that transcripts with main effects of infection (shared across all genotypes) vastly outnumbered transcripts with genotype‐specific responses to infection (Lohman et al., 2017). Here, we report similar results. Only four genes responded differently to infection between the two most disparate crosses (RBC/GBC). Still, these four genes provide some insight regarding mechanisms of resistance, particularity a subset of two which have roles in immunity: CCN family member 3 (CCN3) and SH2 domain‐containing protein 1A (SH2D1A). CCN3 expression suppresses fibrosis responses through the modification of expression of other CCN family proteins (Abd El Kader et al., 2013). CCN3 decreased more significantly in response to infection in resistant (RBC) fish, perhaps contributing to observed fibrosis phenotypes. SH2D1A is an important mediator of humoral immunity, particularly long‐term immune memory, largely through regulation of CD4^+^ T cell functioning (Crotty, 2011; Crotty et al., 2003). Furthermore, SH2D1A may affect NKT cell processes (Nichols et al., 2005; Wu et al., 2016) and differentiation of T_H_2 cells (Wu et al., 2001). Interestingly, expression of this key component of adaptive immunity was maintained in resistant fish (RBC) in response to infection, but significantly decreased in the susceptible cross (GBC), highlighting the importance of adaptive immunity, and potentially long‐term immune memory, in resistance to cestode infection in *G*. *aculeatus*. Thus, while we identified limited signatures of divergent inducible responses to infection among our cross types, those gene which we did identify may be significant contributors to the mechanisms of resistance.

### 4.4 Expression changes associated with fibrosis

In addition to evaluating the roles of variation in *G*, *E*, *G *× *E* responses in divergent parasite responses, our findings also shed light on a putative resistance phenotype in this system: fibrosis. Fibrosis is a common immune pathology across vertebrates (Sgalla et al., 2016; Vrtílek & Bolnick, 2020; Wick et al., 2010), frequently associated with parasitic infections (Kamdem et al., 2018; Niu et al., 2019; Wilson et al., 2007). Often excessive fibrotic responses can cause health issues, including in humans (Henderson et al., 2020; Todd et al., 2012; Wynn, 2008). In *G*. *aculeatus*, recent study has demonstrated that peritoneal fibrosis is an induced response to *S*. *solidus* in some stickleback populations (Hund et al., 2020), and is associated with reduced cestode growth (Weber et al., 2021). More broadly, teleost fish in general are almost all susceptible to peritoneal fibrosis in response to an immune challenge, however little mechanistic knowledge exists regarding the cellular processes controlling these responses (Vrtílek & Bolnick, 2020). Indeed, while the mechanisms connecting fibrosis to systemic immunity in fish remain unclear, our data provides novel insight regarding the broadscale cellular processes linked to the generation of fibrotic tissue. We identified strong transcriptomic signatures of fibrosis. Because we are documenting changes gene expression in the head kidney, an important haematopoietic organ (Kum & Sekki, 2011), rather than the peritoneal tissue itself, it can be assumed these changes are indicative of systemic immunological signatures associated with fibrosis. Many of the genes differentially expressed in fibrotic fish have putative roles in immunity and fibrosis, including 22 genes involved in complement activation. Numerous previous studies have indicated potential cross‐talk between components of the complement cascade and fibrosis responses (Liu et al., 2018; Xavier et al., 2017). Similar to the effects of infection, the majority of these genes were downregulated in fibrotic fish. We also observed 36 collagen genes that were differentially expressed in fibrotic fish, almost all of which were also downregulated. Fibrotic tissue is formed via the deposition of collagen and other extracellular matrix components (Henderson et al., 2020; Wynn, 2008). The large‐scale downregulation of these complement and collagen genes, as well as other immune and fibrosis‐related transcripts, in fibrotic fish may be an artefact of the time of sampling: 42 days after exposure. It is possible that these genes are activated earlier on in the infection time course, but have since been downregulated, potentially as a mechanism for attenuation of fibrosis (Hund et al., 2020). Future time‐course expression studies are needed to clarify the temporal dynamics of gene expression during fibrosis.

**FIGURE 6:**
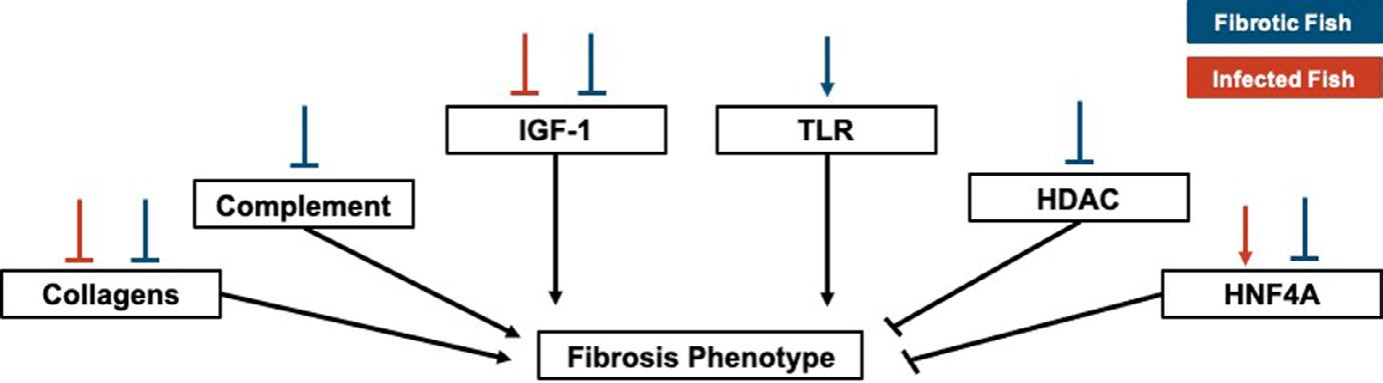
Summary of major changes in gene expression, pathways, and upstream regulators that may affect the resistance phenotype, fibrosis. Lines above each box demonstrate up or down regulation of each component in fibrotic (blue) or infected (red) fish. Lines from each box show the predicted relationship between a given component and the fibrosis phenotype (based on existing literature). In each case arrows indicate upregulation/activation whereas blunt end lines indicate downregulation/inhibition

Pathway and upstream regulator analysis also revealed downregulation of immune‐related process in fibrotic fish, however this downregulation was substantially less than downregulation of the same pathways in infected fish. Many of these pathways differentially expressed in both fibrotic and infected fish have potential ties to fibrotic processes, including hepatic fibrosis signalling pathway and IGF‐1 signalling. IGF‐1 stimulates the differentiation of fibroblasts to promote a potent fibrosis response (Hung et al., 2013). Other pathways uniquely activated in fibrotic fish include the key innate immune component, Toll‐like receptor signalling, which was activated in fibrotic fish. Numerous TLR receptors can bind to parasite antigens to activate an immune response (Ashour, 2015; Coban et al., 2005; Mukherjee et al., 2016), though their links to fibrotic responses are unclear. Finally, pathways related to NFAT regulation of immune response were significantly downregulated in fibrotic fish. NFAT contributes to the regulation of T cell development, diversification, and activation and affect T cell lineage differentiation (i.e., T_H_1 vs. T_H_2; (Lee et al., 2018; Macian, 2005). Downregulation of this pathway suggests fibrotic fish may have reduced regulation of T cell activity, allowing for more potent responses to *S*. *solidus*. In summary, pathway analyses suggest that, compared to infected fish without fibrosis, fibrotic fish have reduced downregulation of immune pathways, and activation of unique prodefence pathways, which may allow for the induction of these putative resistance responses. Upstream regulators show similar patterns. While several key immune regulators were downregulated in both infected and fibrotic fish, fibrotic fish also demonstrated unique signatures of immune response. For example, negative regulator of immunity such as HDAC4 (Yang et al., 2019) was uniquely suppressed in fibrotic fish. Furthermore, antifibrotic regulator hepatic nuclear factor 4‐alpha, HNF4A (Ji et al., 2018; Yue et al., 2010) was significantly activated as a result of infection, but suppressed in fibrotic fish. Combined these results suggest that while infection is marked by a general reduction in immune pathways, this suppression is weaker or absent in fibrotic fish that more effectively reduce cestode growth (Figure 6).

The association between infection and immunosuppression is particularly interesting as immunosuppression is widely known in helminths in general (Maizels et al., 2004, 2018; Maizels & McSorley, 2016), and has been documented for *S*. *solidus* in particular (Scharsack et al., 2004). It is possible the described patterns are indicative of cestode suppression of host immune response during infection. In contrast, fibrotic fish show weaker signatures of immune reduction, and in some instances demonstrate patterns of expression opposite to those displayed by infected, nonfibrotic fish. Furthermore, these specific pathways and genes that demonstrate disparate patterns between fibrotic and infected‐but‐nonfibrotic fish (e.x. HNF4A), are also highly variable among cross‐types. Combined, these analyses suggest that cestodes may act to suppress immune responses in their host, but that this immunosuppression differs between host genotypes, and between fibrotic and nonfibrotic fish. These findings suggest that evolution of resistance may also be dependent on the acquisition of traits to overcome or avoid parasite manipulation of host immunity.

## 5 CONCLUSIONS

Vertebrate immunity is a highly derived, complex system of constitutive and inducible immune responses. Consequently, the effects of selection induced by novel parasites and pathogens can be stochastic and difficult to quantify. Here we provide evidence that divergence in response to parasite selection is a mix of conserved responses and variable inducible responses to the parasite. While we document strong variation in general constitutive expression among cross types, immune defence genes are not statistically overrepresented among these constitutive genetic differences. In contrast we observe a small number of genes closely linked to parasite resistance and immunity which vary in expression in response to infection among our cross types (G × E). The ratio of significantly differentially expressed genes with G, E, and G × E effects is 11,321:2369:569, showing that constitutive genetic divergence is the dominant contribution to expression variation in this system. However, when considering only genes with documented immunological roles (i.e., genes with immune annotations), the ratio is 1216:342:65. Comparing this ratio to the G:E:G × E ratios for all gene annotations, inducible responses (while still a minority) are statistically overrepresented. From this observation we conclude that, at least in this system, inducible responses, as opposed to constitutive defences, contributes disproportionately to variation in immune gene expression. Genotype‐specific immune responses affected a very small minority of genes, though these may nevertheless be functionally quite important. In addition to providing further clarification regarding mechanisms of divergent adaptation to parasites, we also identify transcriptomic signatures contributing to a putative resistance phenotype, fibrosis. Not only do we identify key genes and pathways associated with this response, but we also provide evidence that fibrotic (i.e., resistant) fish apparently are refractory to immune suppression associated with infection in other fish. This suggests host evolution of counter mechanisms may also be key in the evolution of resistance.

## AUTHOR CONTRIBUTIONS

D.I.B., N.C.S. and J.N.W. conceived of and planned the experiments. S.D.H., K.C.S., N.C.S. and J.N.W. conducted the experiments and collected and processed associated samples. L.E.F. analysed gene expression data, conducted correlative analyses and wrote the manuscript. All authors approved of the final manuscript.

## Supporting information

Fig S1‐S2

File S1

File S2

File S3

File S4

## Data Availability

Raw data, including read count matrixes and metadata, and code can be found on GitHub (https://github.com/lfuess/TagSeqMS).
