# Supplementary material for: Between‐population differences in constitutive and infection‐induced gene expression in threespine stickleback
Source: Mol Ecol. 2021 Oct 18;30(24):6791–805. doi: 10.1111/mec.16197 (PMC8796319; doi:10.1111/mec.16197)
Supplement: Supplementary file 1 — Fig S1‐S2 [file MEC-30-6791-s003.docx]

**Supplemental Information for:**

**A test of the Baldwin Effect: Differences in both constitutive expression and inducible responses to parasites underlie variation in host response to a parasite**

Lauren E. Fuess,^,^ Jesse N. Weber, Stijn den Haan, Natalie C. Steinel, Kum Chuan Shim, Daniel I. Bolnick

**Table of Contents:**

| **Figure S1** | Page 1 |
| --- | --- |
| **Figure S2** | Page 2 |
| **File S1–S4** | Page 3 |

**Figure S1**: Venn diagram of overlap in pathways which were differentially activated between each of the three cross types.

**Figure S2:** Predicted patters of activation of upstream regulators which were differentially activated as a result of A) infection and B) fibrosis. C) Comparison of activation (z-score) of upstream regulators that were predicted to be significantly activated/inactivated as a result of both fibrosis and infection. Dotted line represents equivalent activation as a result of both factors; points in blue are more activated in fibrotic fish; points in red are more activated in infected fish.

**Supplementary File Captions:**

**File S1**: List of transcripts which were significantly differentially expressed in infected fish, or between 2 of the host cross types. Data include annotation, log2fold change value, and adjusted p-value for each transcript.

**File S2**: List of pathways which were predicted to be significantly differentially activated in infected fish, or between 2 of the host cross types. Data include adjusted p-value, z-score (metric of activation), and included molecules for each pathway.

**File S3**: List of upstream regulators which were predicted to be significantly differentially activated in infected fish, or between 2 of the host cross types. Data include expression, adjusted p-value, molecule type, predicted activation, z-score (metric of activation), and affected molecules for each regulator.

**File S4**: Genes that were commonly differentially expressed in infected fish across our study and results from a previous study of *G. aculeatus* and *S. solidus* (Haase et al. 2016).
